# RadioImmunotherapy for adenoid cystic carcinoma: a single-institution series of combined treatment with cetuximab

**DOI:** 10.1186/1748-717X-5-102

**Published:** 2010-11-03

**Authors:** Alexandra D Jensen, Jürgen Krauss, Wilko Weichert, Jürgen Debus, Marc W Münter

**Affiliations:** 1 Dept of Radiation Oncology, INF 400, 69120 Heidelberg, Germany; 2 National Centre for Tumour Disease (NCT), INF 460, 69120 Heidelberg, Germany; 3 Institute of Pathology, INF 220/221, 69120 Heidelberg, Germany

## Abstract

**Background:**

Local control in adjuvant/definitive RT of adenoid cystic carcinoma (ACC) is largely dose-dependent. However, some clinical situations do not allow application of tumouricidal doses (i.e. re-irradiation) hence radiation sensitization by exploitation of high endothelial growth factor receptor (EGFR)-expression in ACC seems beneficial. This is a single-institution experience of combined radioimmunotherapy (RIT) with the EGFR-inhibitor cetuximab.

**Methods:**

Between 2006 and 2010, 9 pts received RIT for advanced/recurrent ACC, 5/9 pts as re-irradiation. Baseline characteristics as well as treatment parameters were retrieved to evaluate efficacy and toxicity of the combination regimen were evaluated. Control rates (local/distant) and overall survival were calculated using Kaplan-Meier estimation.

**Results:**

Median dose was 65 Gy, pts received a median of 6 cycles cetuximab. RIT was tolerated well with only one °III mucositis/dysphagia. Overall response/remission rates were high (77,8%); 2-year estimate of local control was 80% hence reaching local control levels comparable to high-dose RT. Progression-free survival (PFS) at 2 years and median overall survival were only 62,5% and 22,2 mo respectively.

**Conclusion:**

While local control and treatment response in RIT seems promising, PFS and overall survival are still hampered by distant failure. The potential benefit of RIT with cetuximab warrants exploration in a prospective controlled clinical trial.

## Introduction

Adenoid cystic carcinomas are rare tumours mostly of the head and neck and account for approximately 10-15% of malignant salivary gland tumours [1]. They are characterised by a rather slow growth pattern but also perineural spread and a high propensity for haematogenous metastases. Standard treatment so far consists of complete surgical resection followed by adjuvant irradiation in case of risk factors (i.e. close margins, perineural invasion, extensive primary tumor (T3, T4) or high-grade histology) [2-4].

Local control in this disease could already be improved by adjuvant radiation, the introduction of high-precision RT techniques (i.e. FSRT and/or IMRT) with consecutive dose escalation, and last but not least high-LET RT. To achieve local control, radiation doses of >60 Gy or even 66 Gy are recommended [5-8].

Initial local control rates combined IMRT plus C12 heavy ion boost to a total dose of 72 GyE were 78% at 4 years [9]. Recent updates including all patients treated at the Gesellschaft für Schwerionenforschung (GSI) Darmstadt between 1997 and 2008 even yielded a local control rate of 82% at 5 years [10,11]. Therefore the combination of IMRT and carbon ion boost shows comparable or even superior control rates to neutron RT [12,13] without increase of late toxicity and subsequent morbidity consistent with outcomes reported by Mizoe et al [14]. Therefore, IMRT plus C12 boost has been accepted as a standard in Germany whenever available.

Albeit progress has been made by the introduction of particle therapy in the treatment concept of adenoid cystic carcinoma, local control rates still leave room for improvement. With the successful introduction of combination regimen in squamous cell carcinoma of the head and neck (SCCHN), leading to a significant improvement not only in local control but also in overall survival, investigation of this approach was obvious in adenoid cystic carcinoma hoping for further improvement of local control and higher response rates of bulky tumours. Radiochemotherapy in the treatment of malignant salivary gland tumors (MSGT) however, has not evolved beyond the phase II-stage or retrospective analysis of very heterogeneous treatment regimen [15-18] into a treatment standard so far as results have been more or less inconclusive.

Immunostaining of surgical specimen however [19], could show over-expression of EGFR in adenoid cystic carcinoma in high percentages hence implying use of targeted therapies as potential alternative [19,20] to comparatively toxic chemotherapy regimen commonly used in recurrent or metastatic adenoid cystic carcinoma since the mid 80-ies [21-24]. Despite the initial euphoria, treatment results have so far failed to impress: no objective response in recurrent or metastatic adenoid cystic carcinoma could be shown in any of the trials [25-27] although prolonged disease stabilization was observed in the reported series [26,27].

Since the publication of combined radioimmunotherapy with the EGF receptor antibody cetuximab in SCCHN of the Bonner trial in 2006 [28,29] though, application of these drugs in adenoid cystic carcinoma seemed feasible in view of potential increase of radiation sensitivity and - albeit modest - systemic activity given the relatively mild toxicity profile of EGFR antibodies. Hence, we would like to present our experiences in combined radioimmunotherapy of adenoid cystic carcinoma with cetuximab.

## Methods

In an individual approach patients received radioimmunotherapy with cetuximab for advanced or recurrent adenoid cystic carcinoma between 01/2006 and 06/2010. Baseline characteristics as well as treatment parameters were retrieved to evaluate efficacy and toxicity of the combination regimen were evaluated.

### Indication

Radioimmunotherapy for adenoidcystic carcinoma not representing a therapeutic standard, medical indication was highly individual and made in interdisciplinary consensus only in cases where applicable radiation doses were deemed insufficient for reasonable tumour control. Given the fact carbon ion treatment was only available three times a year for a very limited number of patients, this series includes patients in need of immediate treatment due to rapid tumour progression or locoregional relapse after prior RT. Sufficient dose prescription (>70 Gy) was not possible in all of these cases either because of proximity/involvement of critical structures or prior RT, hence the idea was to increase efficacy of radiation therapy by combined radioimmunotherapy usually accompanied with only mild toxicity. Rationale for the proposed treatment was extensively discussed and decisions made in accordance with the patients.

5/9 pts hade undergone prior RT (median dose: 58 Gy, range 50,4 - 62 Gy) with a median time interval of 57 months for treatment for adenoidcystic carcinoma. Four pts received radioimmunotherapy as part of their primary treatment but showed extensive tumour mass directly adjacent to or involving critical structures. 8/9 pts received IMRT (2 pts as tomotherapy, 6 pts in step and shoot technique), 1 pt received combined IMRT plus C12-boost due to rapid postoperative local progression.

### Histomorphologic evaluation and immunohistochemistry

**Figure 1:**
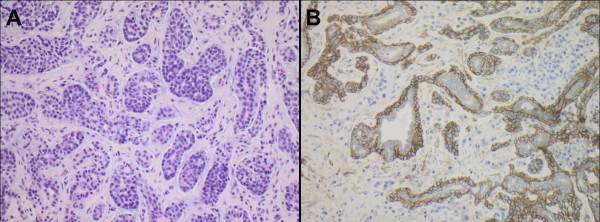
**ACC and EGFR expression**. (A) Histology of an adenoid cystic carcinoma (H&E stain). (B) Strong membranous expression of EGFR in the majority of tumor cells of the same tumor (EGFR immunohistochemistry). Magnification ×200.

The histomorphological diagnosis of adenoid cystic carcinoma was confirmed in all cases by a board certified pathologist with a special expertise in head and neck pathology. For immunohistochemistry 5 ï�­m paraffin sections were cut. Detection of EGFR was performed with the EGFR pharm Dx kit by DAKO (K1492) according to the manufacturer's instructions. Staining was scored as positive if any membranous positivity was observed, however, all investigated cases showed strong membranous positivity in a considerable number of tumor cells (Figure 1a/b).

### Radiation therapy

#### Immobilization/planning examinations

Patients were immobilized using individual scotch cast or thermoplastic head masks with thermoplastic shoulder fixation. Planning examinations consisted of a planning CT scan (3 mm slice thickness) with the patient positioned in the individual fixation device and contrast-enhanced MRI for 3 D image correlation.

#### Target volumes: primary (photon) RT

Target delineation was carried out based on planning CT and MRI scan. CTV1 included the macroscopic tumor/prior tumor bed with a margin of 2 mm with special focus on the R2/R1-area as well as respective neural pathways to the base of skull (cave: perineural invasion and skip lesions).

CTV2 included CTV1 with generous safety margin along typical pathways of spread (if possible safety margin of about 5 cm) depending on the anatomical relationship of adjacent structures. In particular, neurovascular sheaths and locoregional ipsilateral nodal levels were also included in the CTV2.

#### Target volumes: re-irradiation

For patients who had already undergone a course of prior radiotherapy, the treatment volume was strictly limited to the gross tumour volume and did not include elective nodal levels. Doses of were highly individualised but aimed at 50 - 60 Gy re-irradiation in 2 Gy/fraction [30] depending on elapsed time since the first course of RT and prior RT-dose.

#### Target volumes: combined IMRT + carbon ion boost

CTV1 (carbon ion boost) included the macroscopic tumor/prior tumor bed with special focus on the R2/R1-area as well as respective neural pathways to the base of skull (cave: perineural invasion and skip lesions). PTV1 consists of a 3 mm margin around the CTV1 but does not extend into critical organs at risk (i.e. brain stem, spinal cord).

Treatment is given at the HIT (Heidelberg ion therapy centre) after inverse treatment planning in active beam application (raster-scanning method).

CTV2 included CTV1 with safety margins along typical pathways of spread. Only ipsilateral nodal levels (II and III) are included, however, in case the primary tumor is/was located at midline or crossing midline, bilateral nodal levels II and III are covered. In case there is pathological lymph node involvement, additional nodal levels were covered as indicated. CTV2 also encompassed the complete surgical operational area. The CTV2 also takes account for set-up variations, hence corresponds to the PTV2 (CTV2 = PTV2).

#### Immunotherapy

Cetuximab was administered as 400 mg/m2 body surface loading dose 7 days prior to RT-treatment start after administration of anti-histamines (dimetindene) and corticosteroids (dexamethasone).

Weekly administrations of Cetuximab 250 mg/m2 body surface followed for the duration of radiotherapy.

#### Analysis

Treatment response was analysed 6 wks post completion of RIT (first follow-up) and at each available follow-up (best response) according to RECIST criteria [31] based on available follow-up scans (CT or MRI) and clinical examinations. Treatment outcome (locoregional, distant and overall progression-free survival as well as overall survival) was evaluated using higher non-parametric statistics (Kaplan-Meyer survival analysis) with the software xlstat 2010. Progression-free survival was defined as the time from start of combined radioimmunotherapy until the first event (i.e. locoregional relapse, distant metastases, death). Similarly, overall survival was calculated from start of radioimmunotherapy until death from any cause.

## Results

Nine pts with adenoid cystic carcinoma receiving combined radioimmunotherapy with cetuximab were identified. Median follow-up is 12,5 months [1,2 - 29,6 mo]. Tumours were mostly located near or at the base of skull (epipharynx, pterygopalatine fossa, skull base). All patients had a macroscopically visible tumour mass, all but one T4 tumours (patient characteristics see table 1). EGFR expression analysis was available in 8/9 pts (all of them with at least moderate or high expression rates), specimen for one pt were unfit for EGFR analysis.

**Table 1:** patient characteristics

patient characteristics		
**median age**	56 a	[40 - 77]

**tumour localisation**		

	Epipharynx	2 pts

	base of skull	2 pts

	Fossa pterygopalatina	3 pts

	Maxilla	1 pt

	tuba auditiva	1 pt

**tumour stage**	T4	8 pts

	T3	1 pt

	no nodal involvement	9 pts

Median dose applied was 65 Gy (total) and 50,4 Gy in pts receiving the second course of radiation (median cumulative dose 111,2 Gy) after a median interval of 63,7 months between the two courses (table 2). All pts in this series received IMRT either as single modality (8/9pts) or as combined treatment with carbon ion boost (1/9 pts). Prior RT in the 5 cases with re-irradiation was carried out using IMRT (3 pts), stereotactic single fraction only (1 pt) and 3 D conventional (1 pt). All patients completed the treatment as planned; there were no treatment interruptions. A median of 6 cycles cetuximab [4 - 8 cycles] (excluding loading dose) were applied. Treatment was tolerated well without any cases of allergic reactions and only one case of CTC °III toxicity (mucositis °III leading to temporary feeding tube dependence). 8/9pts developed acneiforme skin reactions °I/II, 7/9 pts radiodermatitis °I/II and mucositis °I/II (table 3). In the 7 pts with follow-up available, acute reactions were completely resolved at first follow-up, there were no treatment-related late effects.

**Table 2:** treatment

radiotherapy	median	min	max
**(overall)**			

median dose	65 Gy	39,6 Gy	72,8 Gy

**(re-RT)**			

median dose	50,4 Gy	39,6 Gy	69,9 Gy

median cumulative dose	111,2 Gy	97,6 Gy	130,5 Gy

median interval between RTs	63,7 mo	11,3 mo	91,1 mo

**Table 3:** observed toxicity

acute toxicity	CTC v3.0		
	**I/pts**	**II/pts**	**III/pts**

**acneiforme dermatitis**	4	4	

**radiogenic erythema**	5	2	

**mucositis**	2	5	1

**dysphagia**	3	0	1

**xerostomia**	2		

**Figure 2:**
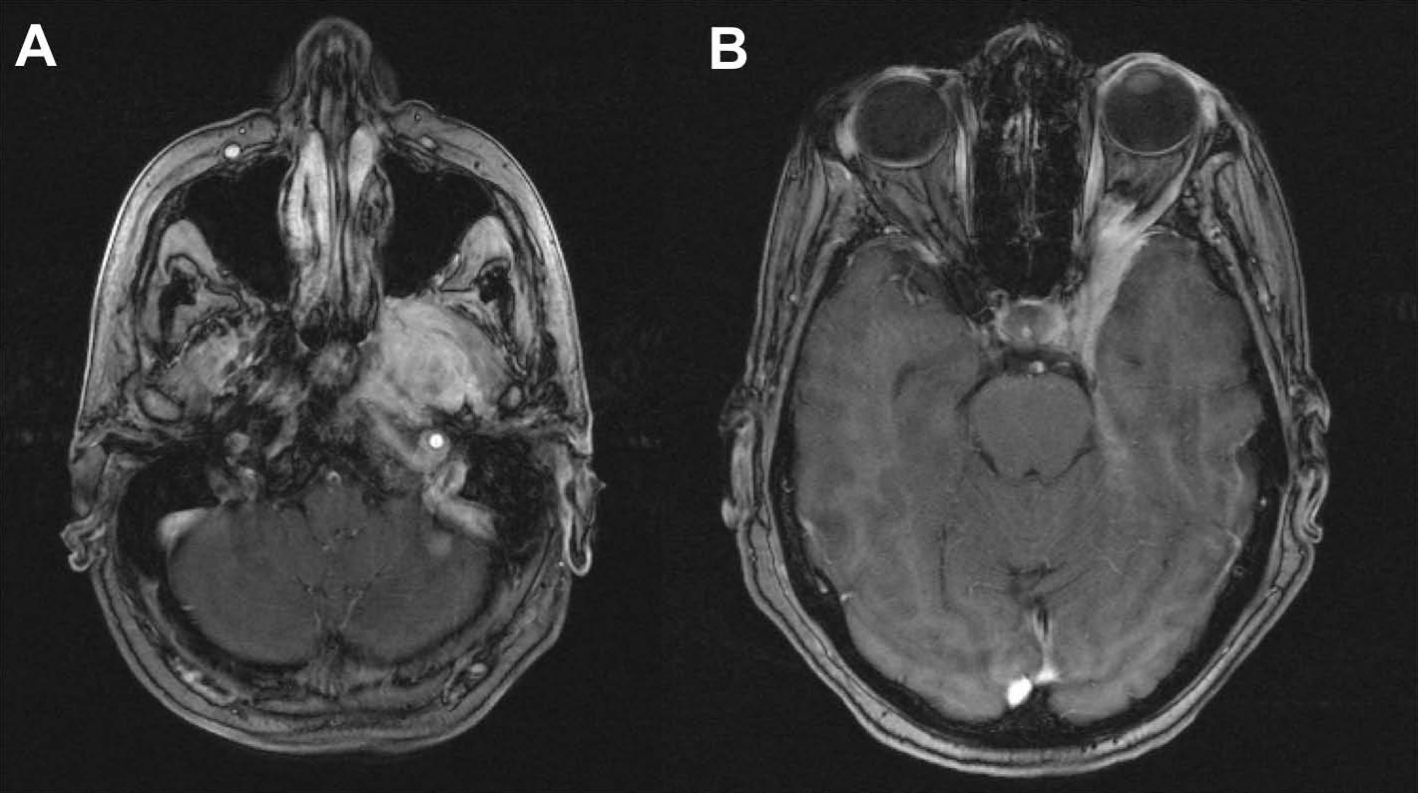
**Initial, contrast-enhanced MRI of a large adenoid cystic carcinoma extending from the left pterygoid muscles (a) into the cavernous sinus and left orbit (b)**.

**Figure 3:**
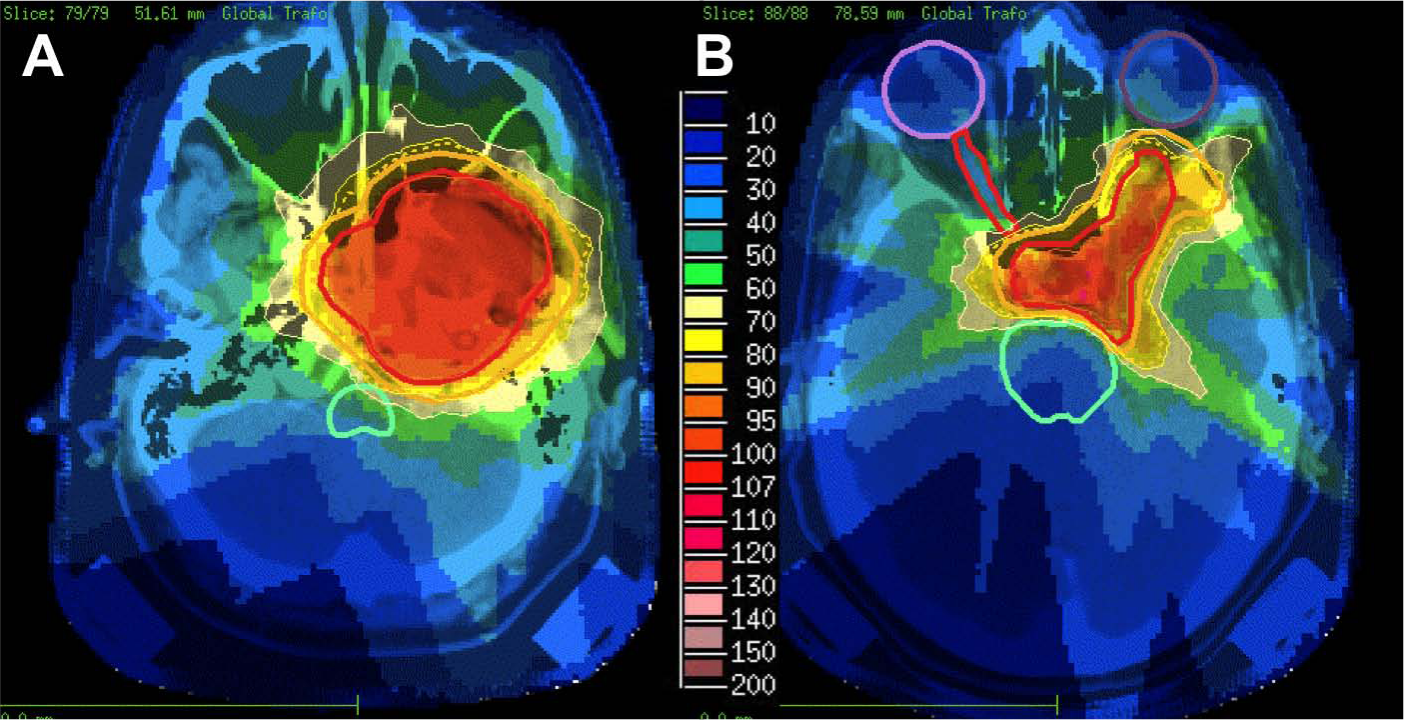
**IMRT treatment plan applying an integrated boost concept; dose distribution pterygoid muscles (a) and cavernous sinus/left orbit (b); 100% corresponding to 66 Gy; CTV1 receives a median of 66 Gy, CTV2 was prescribed 54 Gy**.

At first follow-up 8/9 pts (88,9%) showed good partial remissions. 1 pt died prior to the first f/u (6 wks post RT) due to tumor bleeding. (table 4). On further f/u, 6/7pts stayed locally controlled. Figure 2(a/b) shows the pretherapeutic MRI scan for a patient with large ACC extending from the optic canal to the pterygoid muscles. The applied IMRT treatment plan using a simultaneous integrated boost concept and the corresponding DVH are shown in Figures 3a/b and 4. 66 Gy were prescribed to the median of CTV1. However, impaired coverage needed to be accepted in order to spare the left optic nerve. This patient is still locally controlled 21/2 years post treatment, one f/u scan showing good PR is depicted on Figure 5 (a/b). Another pt actually showed a complete remission on further f/u corresponding to a 2-year local control rate of 80% (Figure 6). 1 pt developed a regional relapse outside the re-RT field 10 mo post re-RT, 1 pt developed local, locoregional, and distant relapse (pulmonary and hepatic metastases). Another patient has so far stayed locally controlled but did develop bone metastases, distant control and PFS at 2 years are 62,5% (Figures 7+8). Four pts received further treatment (2 pts with chemotherapy for local progression (carboplatin/vinorelbine, 1 pt) and distant failure (paclitaxel, 1 pt), 1 pt received photodynamic therapy for contralateral progression (out-of field), 1 pt received further radiation for bone metastases). Four patients are deceased as of July 2010 due to disease progression, hence overall survival at 2 years is only 25% and corresponding to disease-specific survival (Figure 9).

**Table 4:** treatment response

treatment response		@ 6-8 wks post Rt	further f/u
**total**	PR	7 pts	6 pts

	CR		1 pt

	dna	2 pts	2 pts

**re-RT**	PR	4 pts	2 pts

	CR	0	1 pt

	PD	0	1 pt

	dna	1 pt	1 pt

**Figure 4:**
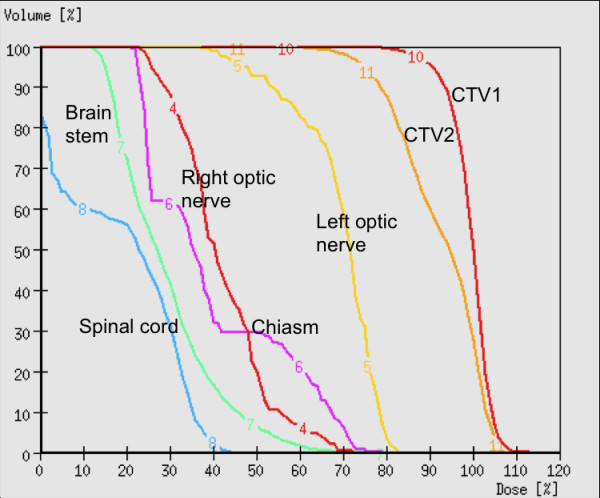
**Corresponding DVH; dose prescribed to the median of CTV1; 100% := 66 Gy**.

**Figure 5:**
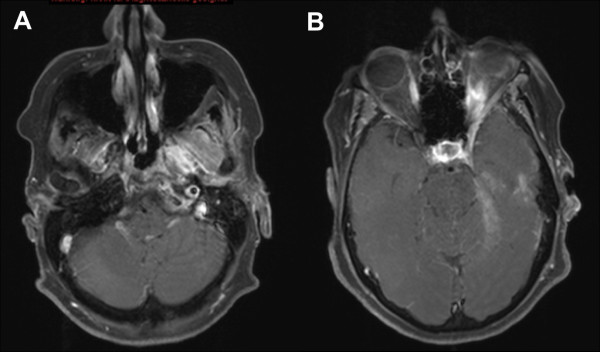
**Follow-up contrast enhanced MRI 14 months post radioimmunotherapy: therapy-related changes: pterygoid muscles (a) and cavernous sinus/left orbit (b)**.

**Figure 6:**
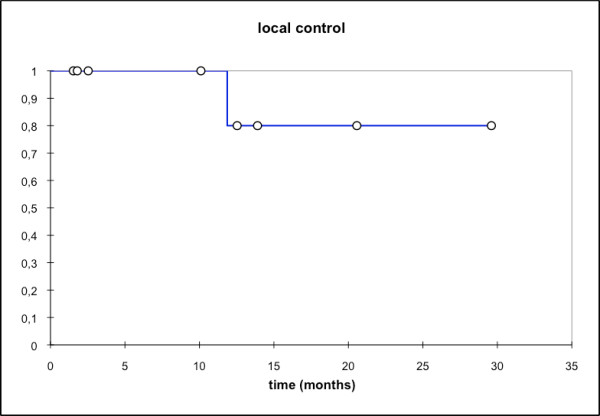
**Local control; mean local ctrl: 13,5 mo; local ctrl @2a:80%**.

**Figure 7:**
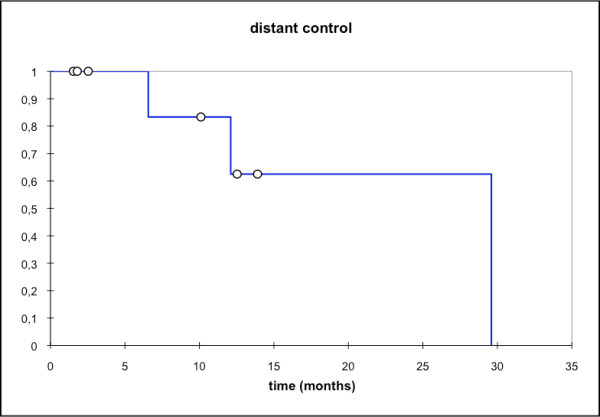
**Distant control; mean distant ctrl**. 22,1 mo; median distant ctrl: 29,6 mo [95% CI: 12,1 - 29,6 mo].

**Figure 8:**
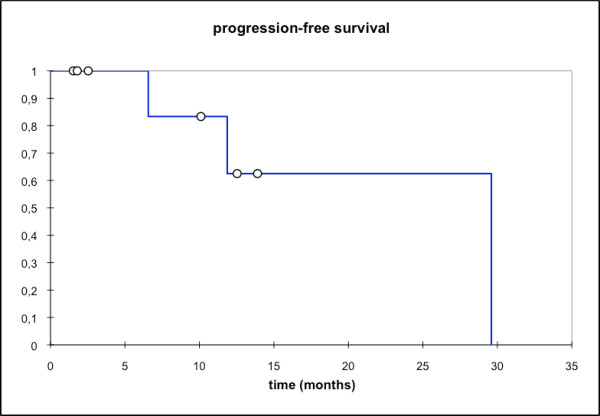
**overall progression-free survival (PFS); mean PFS: 22,1 mo, median PFS: 29,6 mo [95% CI: 11,9 - 29,6 mo]; PFS @ 2a: 62,5%**.

**Figure 9:**
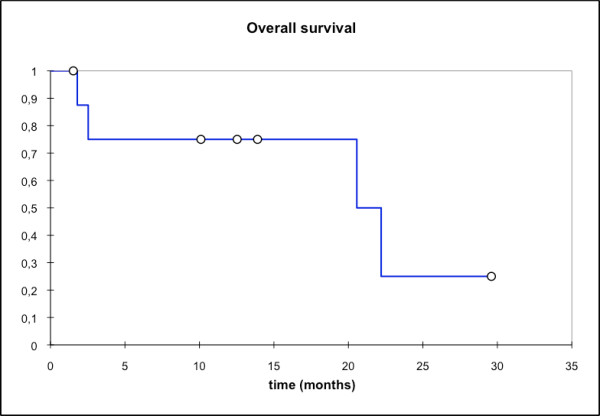
**Overall survival; mean OS: 16,8 mo; median OS: 22,2 mo; OS @2a: 25%**.

## Discussion

In SCCHN, combination therapy with the EGFR-antibody cetuximab yielded comparable results to chemoradiation regimen on retrospective comparisons [29,32] without increase of toxicity - except acneiforme skin reactions - and has therefore raised interest for this combination also in other histologies. Analyses on adenoid cystic carcinoma surgical specimen showed high rates of EGFR and c-kit expression, hence potential targets for biological agents [19,20]. Faced with limitations in dose prescription in the presented cases, we saw the chance to exploit radio-sensitizing potential of the EGFR- antibody cetuximab to potentially improve these patients' outcome despite necessary compromises on dose and target volume coverage at the cost of very low toxicity. Consistent with the data by Bonner et al [28,29,32], combined radioimmunotherapy with cetuximab was tolerated well and without any major, treatment-related side effects despite one case of °III mucositis and consecutive dysphagia. Considering the extent and localisation of the target volume, this would also have been expected in single-modality RT.

Treatment response with approx. 78% in all pts (7/9 pts) and 100% in pts with follow-up available as well as local control (80% @2a) were comparatively high despite the fact 5/9 pts (55,6%) received therapy as re-irradiation. So far, published data report local control rates in adenoid cystic carcinoma of 78% at 2 and 4 years with IMRT and carbon ion boost [9], between 75 - 100% for particle therapy including neutrons [12,13,33], and between 24,5% and 82% in at least R1 resected tumours with up to 62% in large primary or R2-resected disease [4,7,13,34]. While our median local control has not been reached, the actuarial local control in our series was 80% at 2 years, hence slightly higher than in the previously published IMRT-series (local control of 75% at 2 years and 38% at 4 years [34]) and comparable to the above mentioned carbon ion results [9]. Considering a median overall survival of 20 months, it is, however, not possible to extrapolate expected local control rates. As reported by the groups of Chen and Garden [6-8], local control was largely dependent on applied dose, hence doses of >60-66 Gy are recommended. Despite necessary compromises on target volume coverage and or dosage, all of our patients showed at least a partial remission on their follow-up scans, hence, response rates of the combined approach are encouraging.

While we have only seen one case of local failure in this cohort, distant failure seemed much higher (distant ctrl @2a: 62,5%), hence the progression-free survival rate is largely influenced by the rate of distant failure. Overall survival in this series with 25% at 2 years was disappointing, however, none of the pts in previous series had undergone re-RT or been treated for disease recurrence, therefore worse outcomes might be expected with patients already having a long history of their disease. Overall survival being a function of distant failure is a finding consistent with our previous experience and other groups [9,12,13,34-36], therefore, a lot of research has been directed at improvement of systemic control in adenoidcystic carcinoma. Faced with the sometimes very slow progression, interest in targeted therapies for systemic treatment of MSGT and accompanying mild toxicity profile arose very early on. Based on pathological findings of high EGFR-expression in adenoid cystic carcinoma surgical specimen [19], there have been various phase I-II trials using EGFR antibodies or tyrosine kinase inhibitors in locally recurrent/metastatic pts [25-27]. However, though prolonged disease stabilization was observed, no objective response could be shown. It might be worth noting that initial experiments with cetuximab and radiation in various cell lines were in fact able to establish a synergistic effect of the two treatment components resulting in increased efficiency of the combination than either single modality [37,38]. Hence, the fact cetuximab might not have vast impact in metastatic disease does not preclude efficiency of the combination regimen. Further individualization of treatment will require identification of predictors for metastatic spread in adenoid cystic carcinoma in order to intensify the systemic treatment component for these patients. Patients with localized disease however, may profit from more aggressive local treatment procedures such as combined with carbon ion therapy as proposed here.

## Conclusion

In Summary, radioimmunotherapy with cetuximab was tolerated well and yielded promising response and local control rates. Overall survival in this series was comparatively low and it remains unclear whether cetuximab or any other EGFR-antibody/tyrosine kinase inhibitor may reduce the rate of distant metastases.

Hence, a prospective controlled trial is needed to investigate the potential significance of targeted therapies/combined radioimmunotherapy for EGFR positive adenoid cystic carcinomas in a representative, homogeneous and untreated patient cohort. The ACCEPT trial (**A**denoid**c**ystic **c**arcinoma, **E**rbitux^® ^and **p**article **t**herapy) is currently in preparation to answer these questions.

## Conflict of interest

JD is am member of Merck KGa advisory board.

## Authors' contributions

ADJ, JK, WW, JD, and MWM were responsible for individual treatments, concepts, and decisions; WW performed histopathological investigation of tissue samples.

All authors read and approved the final manuscript.

## References

[B1] Spiro RH Salivary neoplasms: overview of a 35-year experience with 2,807 patients Head Neck Surg 1986 8 3 177 84 10.1002/hed.2890080309 3744850

[B2] Chen AM Granchi PJ Garcia J Bucci MK Fu KK Eisele DW Local-regional recurrence after surgery without postoperative irradiation for carciomas of the major salivary glands: implications for adjuvant therapy Int J Radiat Oncol Biol Phys 2007 67 982 987 17241753 10.1016/j.ijrobp.2006.10.043

[B3] Gurney TA Eisele DW Weinberg V Shin E Lee N Adenoid cystic carcinoma of the major salivary glands treated with surgery and radiation Laryngoscope 2005 115 7 1278 82 10.1097/01.MLG.0000165381.64157.AD 15995521

[B4] Mendenhall WM Morris CG Amdur RJ Werning JW Hinerman RW Villaret DB Radiotherapy alone or combined with surgery for adenoid cystic carcinoma of the head and neck Head Neck 2004 26 2 154 62 10.1002/hed.10380 14762884

[B5] Chen AM Bucci MK Weinberg V Garcia J Quivey JM Schechter NR Phillips TL Fu KK Eisele DW Adenoid cystic carcinoma of the head and neck treated by surgery with or without postoperative radiation therapy: prognostic features of recurrence Int J Radiat Oncol Biol Phys 2006 66 1 152 9 16904520 10.1016/j.ijrobp.2006.04.014

[B6] Garden AS Weber RS Ang KK Morrison WH Matre J Peters LJ Postoperative radiation therapy for malignant tumors of minor salivary glands. Outcome and patterns of failure Cancer 1994 73 10 2563 9 10.1002/1097-0142(19940515)73:10<2563::AID-CNCR2820731018>3.0.CO;2-X 8174054

[B7] Chen AM Bucci MK Quivey JM Garcia J Eisele DW Fu KK Long-term outcome of patients treated by radiation therapy alone for salivary gland carcinomas Int J Radiat Oncol Biol Phys 2006 66 1044 1050 16965870 10.1016/j.ijrobp.2006.06.050

[B8] Terhaard CH Lubsen H Rasch CR Levendag PC Kaanders HH Tjho-Heslinga RE van Den Ende PL Burlage F Dutch Head and Neck Oncology Cooperative Group. The role of radiotherapy in the treatment of malignant salivary gland tumors Int J Radiat Oncol Biol Phys 2005 61 103 111 15629600 10.1016/j.ijrobp.2004.03.018

[B9] Schulz-Ertner D Nikoghosyan A Didinger B Münter M Jäkel O Karger CP Debus J Therapy strategies for locally advanced adenoid cystic carcinomas using modern radiation therapy techniques Cancer 2005 104 2 338 44 10.1002/cncr.21158 15937907

[B10] Münter M Umathum V Nikoghosyan A Jensen A Hof H Jaekel O Debus J Combination of intensity modulated radiation therapy (IMRT) and a carbon ion boost for subtotal resected or inoperable adenoid cystic carcinomas (ACC's) of the head and neck PTCOG meeting 2009 abstract FC84

[B11] Umathum V Jensen A Nikoghosyan A Hof H Jaekel O Debus J Münter MW Intensitätsmodulierte Radiotherapie (IMRT) in Kombination mit einem Kohlenstoffionenbosst (C-12) bei adenoidzystischen Karzinomen (ACCs) der Kopf-/Halsregionen: Prognostischer Vergleich zwischen resezierten und nicht-resezierten Patienten DEGRO meeting 2010 abstract W19-04. http://degro.wcenter.de/dav/html/kongress2010/eposter/W19-04.pdf as of 30.06.2010

[B12] Huber PE Debus J Latz D Zierhut D Bischof M Wannenmacher M Engenhart-Cabillic R Radiotherapy for advanced adenoid cystic carcinoma: neutrons, photons or mixed beam? Radiother Oncol 2001 59 2 161 7 10.1016/S0167-8140(00)00273-5 11325445

[B13] Douglas JG Koh WJ Austin-Seymour M Laramore GE Treatment of salivary gland neoplasms with fast neutron radiotherapy Arch Otolaryngol Head Neck Surg 2003 129 9 944 8 10.1001/archotol.129.9.944 12975266

[B14] Mizoe JE Tsujii H Kamada T Matsuoka Y Tsuji H Osaka Y Hasegawa A Yamamoto N Ebihara S Konno A Organizing Committee for the Working Group for Head-And-Neck Cancer. Dose escalation study of carbon ion radiotherapy for locally advanced head-and-neck cancer Int J Radiat Oncol Biol Phys 2004 60 2 358 64 15380567 10.1016/j.ijrobp.2004.02.067

[B15] Haddad RI Posner MR Busse PM Norris CM Goguen LA Wirth LJ Blinder R Krane JF Tishler RB Chemoradiotherapy for adenoid cystic carcinoma: preliminary results of an organ sparing approach Am J Clin Oncol 2006 29 153 157 10.1097/01.coc.0000203756.36866.17 16601434

[B16] Airoldi M Pedani F Marchionatti S Gabriele AM Succo G Gabriele P Bumma C Concomitant chemoradiotherapy followed by adjuvant chemotherapy in parotid gland undifferentiated carcinoma Tumori 2001 87 14 17 11669550 10.1177/030089160108700103

[B17] Tanvetyanon T Qin D Padhya T McCaffrey J Zhu W Boulware D DeConti R Trotti A Outcomes of postoperative concurrent chemoradiotherapy for locally advanced major salivary gland carcinoma Arch Otolaryngol Head Neck Surg 2009 135 687 692 10.1001/archoto.2009.70 19620591

[B18] Pederson AW Haraf DJ Blair EA Stenson KM Witt ME Vokes EE Salama JK Chemoreirradiation for recurrent salivary gland malignancies Radiother Oncol 2010 95 308 311 10.1016/j.radonc.2010.03.006 20385414

[B19] Vered M Braunstein E Buchner A Immunhistochemical study of epidermal growth factor receptor in adenoid cystic carcinoma of salivary gland origin Head Neck 2002 24 632 636 10.1002/hed.10104 12112535

[B20] Younes MN Park YW Yazici YD Gu M Santillan AA Nong X Kim S Jasser SA El-Naggar AK Myers JN Concomitant inhibition of epidermal growth factor receptor tyrosine kinases reduces growth and metastasis of human salivary adenoid cystic carcinoma on an orthotopic nude mouse model Mol Cancer Ther 2006 5 2696 2705 10.1158/1535-7163.MCT-05-0228 17121916

[B21] De Haan LD de Mulder PH Vermorken JB Schornagel JH Vermey A Verweij J Cisplatin-based chemotherapy in advanced adenoid cystic carcinoma of the head and neck Head Neck 1992 14 273 277 10.1002/hed.2880140403 1381339

[B22] Creagan ET Woods JE Rubin J Schaid DJ Cisplatin-based chemotherapy for neoplasms arising from salivary glands and contiguous structures in the head and neck Cancer 1988 62 2313 2319 10.1002/1097-0142(19881201)62:11<2313::AID-CNCR2820621110>3.0.CO;2-4 3179947

[B23] Dreyfuss AI Clark JR Fallon BG Posner MR Norris CM Miller D Cyclophosphamide, doxorubicin, and cisplatin combination chemotherapy for advanced carcinomas of salivary gland origin Cancer 1987 60 2869 2872 10.1002/1097-0142(19871215)60:12<2869::AID-CNCR2820601203>3.0.CO;2-Y 2824016

[B24] Venook AP Tseng A Meyers FJ Silverberg I Boles R Fu KK Cisplatin, doxorubicin, and 5-fluorouracil chemotherapy for salivary gland malignancies: a pilot study of the Northern California Oncology Group J Clin Oncol 1987 5 951 955 3585449 10.1200/JCO.1987.5.6.951

[B25] Hotte SJ Winquist EW Lamont E MacKenzie M Vokes E Chen EX Brown S Pond GR Murgo A Siu LL Imatinib mesylate in patients with adenoid cystic cancers of the salivary glands expressing c-kit: a Princess Margaret Hospital Phase II Consortium Study J Clin Oncol 2005 23 585 590 10.1200/JCO.2005.06.125 15659505

[B26] Locati LD Bossi P Perrone F Potepan P Crippa F Mariani L Casieri P Orsenigo M Losa M Bergamini C Liberatoscioli C Quattrone P Calderone RG Rinaldi G Pilotti S Licitra L Cetuximab in recurrent and/or metastatic salivary gland carcinomas: a phase II study Oral Oncology 2009 45 574 578 10.1016/j.oraloncology.2008.07.010 18804410

[B27] Agulnik M Cohen EW Cohen RB Chen EX Vokes EE Hotte SJ Winquist E Laurie S Hayes DN Dancey JE Brown S Pond GR Lorimer I Daneshmand M Ho J Tsao MS Siu LL Phase II study of lapatinib in recurrent or metastatic epidermal growth fator receptor and/or erbB2 expressing adenoid cystic carcinoma and non-adenoid cystic carcinoma malignant tumors of the salivary glands J Clin Oncol 2007 25 3978 3984 10.1200/JCO.2007.11.8612 17761983

[B28] Bonner JA Harari PM Giralt J Azarnia N Shin DM Cohen RB Jones CU Sur R Raben D Jassem J Ove R Kies MS Baselga J Youssoufian H Amellal N Rowinsky EK Ang KK Radiotherapy plus cetuximab for squamous-cell carcinoma of the head and neck N Engl J Med 2006 354 567 578 10.1056/NEJMoa053422 16467544

[B29] Bonner JA Harari PM Giralt J Cohen RB Jones CU Sur RK Raben D Baselga J Spencer SA Zhu J Youssoufian H Rowinsky EK Ang KK Radiotherapy plus cetuximab for locoregionally advanced head and neck cancer: 5-year survival data from a phase 3 randomised trial, and relation between cetuximab-induced rash and survival Lancet Oncol 2010 11 21 28 10.1016/S1470-2045(09)70311-0 19897418

[B30] Janot F de Raucourt D Benhamou E Ferron C Dolivet G Bensadoun RJ Hamoir M Géry B Julieron M Castaing M Bardet E Grégoire V Bourhis J Randomized trial of postoperative reirradiation combined with chemotherapy after salvage surgery compared with salvage surgery alone in head and neck carcinoma J Clin Oncol 2008 26 5518 5523 10.1200/JCO.2007.15.0102 18936479

[B31] Therasse P Arbuck SG Eisenhauer EA Wanders J Kaplan RS Rubinstein L Verweij J Van Glabbeke M van Oosterom AT Christian MC Gwyther SG New guidelines to evaluate the response to treatment in solid tumors J Natl Cancer Inst 2000 92 205 216 10.1093/jnci/92.3.205 10655437

[B32] Curran D Giralt J Harari PM Ang KK Cohen RB Kies MS Jassem J Baselga J Rowinsky EK Amellal N Comte S Bonner JA Quality of life in head and neck cancer patients after treatment with high-dose radiotherapy alone or in combination with cetuximab J Clin Oncol 2007 25 2191 2197 10.1200/JCO.2006.08.8005 17538164

[B33] Pommier P Liebsch NJ Deschler DG Lin DT McIntyre JF Barker FG Adams JA Lopes VV Varvares M Loeffler JS Chan AW Proton beam radiation therapy for skull base adenoid cystic carcinoma Arch Otolaryngol Head Neck Surg 2006 132 11 1242 9 10.1001/archotol.132.11.1242 17116822

[B34] Münter MW Schulz-Ertner D Hof H Nikoghosyan A Jensen A Nill S Huber P Debus J Inverse planned stereotactic intensity modulated radiotherapy (IMRT) in the treatment of incompletely and completely resected adenoid cystic carcinomas of the head and neck: initial clinical results and toxicity of treatment Radiat Oncol 2006 1 17 10.1186/1748-717X-1-17 16756669 PMC1550720

[B35] Gomez DR Hoppe BS Wolden SL Zhung JE Patel SG Kraus DH Shah JP Ghossein RA Lee NY Outcomes and prognostic variables in adenoid cystic carcinoma of the head and neck: a recent experience Int J Radiat Oncol Biol Phys 2008 70 1365 1372 18029108 10.1016/j.ijrobp.2007.08.008

[B36] Lloyd S Yu JB Wilson LD Decker RH Determinants and patterns of survival in adenoid cystic carcinoma of the head and neck, including analysis of adjuvant radiation therapy Am J Clin Oncol 2010 in press 20177363 10.1097/COC.0b013e3181d26d45

[B37] Huang SM Bock JM Harari PM Epidermal growth factor receptor blockade with C225 modulates proliferation, apoptosis, and radiosensitivity in squamous cell carcinoma of the head and neck Cancer Res 1999 59 1935 1940 10213503

[B38] Saleh MN Raisch KP Stackhouse MA Grizzle WE Bonner JA Mayo MS Kim HG Meredith RF Wheeler RH Buchsbaum DJ Combined modality therapy of A431 human epidermoid cancer using anti-EGFR antibody C225 and radiation Cancer Biother Radiopharm 1999 14 451 463 10.1089/cbr.1999.14.451 10850332

